# The impact of heart, lung and diaphragmatic ultrasound on prediction of failed extubation from mechanical ventilation in critically ill patients: a prospective observational pilot study

**DOI:** 10.1186/s13089-018-0096-1

**Published:** 2018-07-04

**Authors:** Kavi Haji, Darsim Haji, David J. Canty, Alistair G. Royse, Cameron Green, Colin F. Royse

**Affiliations:** 1 Department of the Intensive Care Unit, Frankton Hospital, PO Box 52, Frankston, VIC 3199 Australia; 2 Faculty of Medicine, Nursing and Health Sciences, Monash University, Melbourne, Australia; 3 Frankston Hospital, Frankston, VIC Australia; 4 Department of Surgery, University of Melbourne, Melbourne, Australia; 5 Department of Medicine, Health Sciences and Nursing, Monash University, Melbourne, Australia; 6 Royal Melbourne Hospital, Melbourne, Australia; 7 Ultrasound Education Group, Department of Surgery, The University of Melbourne, Melbourne, Australia; 8 Department of Cardiothoracic Surgery, The Royal Melbourne Hospital, Melbourne, Australia; 9 Department of Anaesthesia and Pain Management, The Royal Melbourne Hospital, Melbourne, VIC Australia

**Keywords:** Weaning, Echocardiography, Ultrasound, Lung, Diaphragm, RSBI

## Abstract

**Background:**

Failed extubation from mechanical ventilation in critically ill patients is multifactorial, complex and not well understood. We aimed to identify whether combined transthoracic echocardiography, lung and diaphragmatic ultrasound can predict extubation failure in critically ill patients.

**Results:**

Fifty-three participants who were intubated > 48 h and deemed by the treating intensivist ready for extubation underwent a 60-min pre-extubation weaning trial (pressure support ≤ 10 cmH_2_O and positive end expiratory pressure 5 cmH_2_O). Prior to extubation, data collected included ultrasound assessment of left ventricular ejection fraction, left atrial area, early diastolic trans-mitral flow velocity wave (*E*), early diastolic trans-mitral flow velocity wave/late diastolic trans-mitral flow velocity wave (*E*/*A*), early diastolic trans-mitral flow velocity wave/early diastolic mitral annulus velocity (*E*/*E*′), interatrial septal motion, lung loss of aeration score and diaphragm movement. At the end of the weaning trial, the rapid shallow breathing index and serum B-type natriuretic peptide concentration were measured. Success and failure of weaning was assessed by defined criteria. Decision to extubate was at the discretion of the treating intensivist. Failure of extubation was defined as re-intubation, non-invasive ventilation or death within 48 h after extubation. Of 53 extubated participants, 11 failed extubation. Failed extubation was associated with diabetes, ischaemic heart disease, higher *E/E′* (OR 1.27, 95% CI 1.05–1.54), left atrial area (OR 1.14, CI 1.02–1.28), fixed rightward curvature of the interatrial septum (OR 12.95, CI 2.73–61.41), and higher loss of aeration score of anterior and lateral regions of the lungs (OR 1.41, CI 1.01–1.82).

**Conclusions:**

Failed extubation in mechanically ventilated patients is more prevalent if markers of left ventricular diastolic dysfunction and loss of lung aeration are present.

## Background

Weaning failure is defined as failing a spontaneous breathing trial or developing a post-extubation respiratory distress that requires re-intubation or non-invasive ventilation within 48 h following extubation [1]. Weaning failure is common and time consuming with approximately 40% of the total ventilation time in “difficult to wean” patients is devoted to weaning [2]. Weaning failure is associated with worse outcome [3], increased risk of myocardial ischaemia [4] and perhaps psychological trauma [5].

Identification of reliable predictors of weaning failure may represent potential avenues of treatment that could reduce the incidence of weaning failure and its associated morbidity. Unfortunately, the pathophysiology of weaning failure is complex and is incompletely understood. Known risk factors of weaning failure have considerable crossover, especially those related to the heart, lungs and diaphragm. Weaning is comparable to a cardiac stress exercise where it is likely to increase the heart rate, the pulmonary artery occlusion pressure, which results in myocardial stretch and may increase cardiac output [6]. The myocardial stretch is evidenced by increased release of neurohormone, B-type natriuretic peptide (BNP), which therefore has been suggested as a useful predictor of weaning failure [7]. Known predictors of weaning failure include chronic obstructive airway disease [1], cardiac failure [8–10], lung de-recruitment [11], positive fluid balance [12], pneumonia [12] and diaphragmatic dysfunction [13]. Rapid shallow breathing index (RSBI) is a clinical predictor of failure of weaning from mechanical ventilation and it is widely used in clinical research and in practice [14].

Ultrasound of multiple organ systems is becoming more commonplace in the intensive care unit (ICU) setting. Ultrasound has been used to identify cardiac, respiratory or diaphragmatic risk factors of weaning failure [8, 11, 13]. Further, ultrasound may be helpful in providing a visual assessment of cardiorespiratory state at different phases of weaning [15]. Therefore, a combined structured transthoracic echocardiogram (TTE), lung and diaphragm ultrasound examination added to conventional clinical predictors could be a useful tool to increase the accuracy in predicting weaning outcome and extubation failure. Currently, there are limited data investigating this.

The primary aim of this pilot study was to determine which ultrasound, or clinical and biochemical indicators, were associated with weaning failure. Data from this pilot study will be used to construct future clinical trials.

## Methods

### Study design and setting

This single-centre, prospective observational pilot study was conducted at a 15-bed, level-III ICU that receives approximately 1500 admissions of medical and surgical presentations per year.

### Participants

Recruitment was performed between June 2014 and April 2016, when the primary researcher was available (convenience sampling). Participants aged 18 years or older who were mechanically ventilated for longer than 48 h and considered ready for extubation by the treating intensivist were considered eligible for recruitment. Participants were excluded if they had a diagnosis of brain death, were ventilating through a tracheostomy or if extubation was for palliation. Written informed consent was obtained from the person responsible for decision-making on behalf of the patient.

### Summary of study conduct

**Fig. 1:**
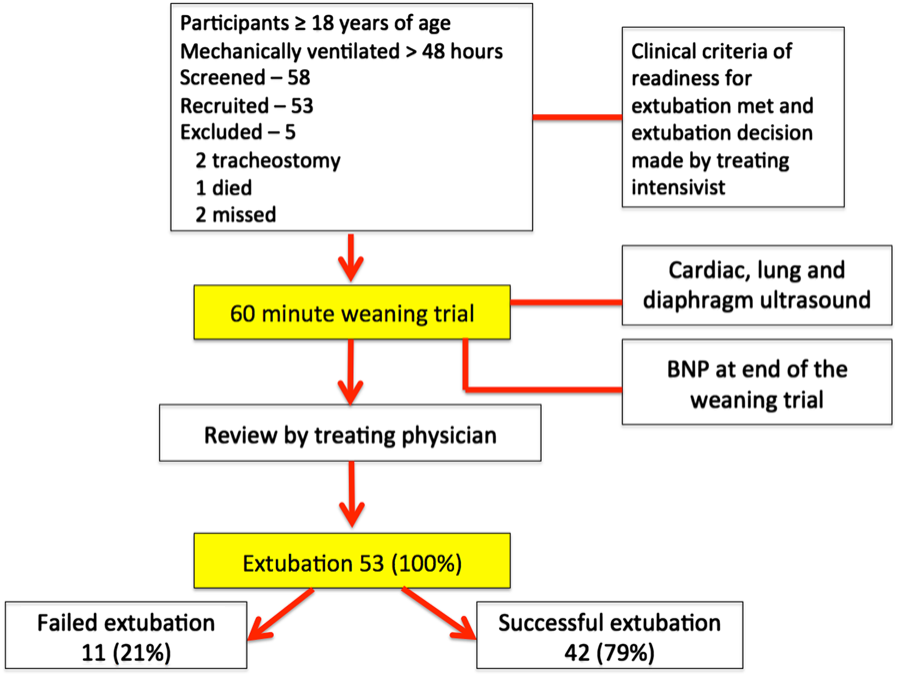
The participants’ flow diagram

Participants were considered eligible for inclusion when participants fulfilled the criteria for weaning from mechanical ventilation defined below and authorized by the treating intensivist (Fig. 1). After informed written consent obtained, a 60-min weaning trial was commenced. The primary researcher (KH) performed and documented ultrasound findings of the heart, lungs and diaphragm as defined below, at the beginning of the weaning trial. At the end of the weaning trial, a sample of arterial blood was collected for blood gas analysis and for BNP measurement (The iSTAT Point of Care Analyzer, Abbott Point of Care Inc., Princeton, Ontario, Canada). The primary endpoint of the study was failure of extubation, which was defined as re-intubation or non-invasive ventilation within 48 h after extubation.

### Weaning trial

Pressure support is a spontaneous mode of ventilation where the patient breathes spontaneously and the ventilator supports the breathing with a preset pressure value for every breath. Decremented pressure support ventilation is the standard mode of weaning in our unit. Extubation at pressure support up to 10 cmH_2_O is an acceptable practice in our unit and a spontaneous breathing trial using T-tube is not mandated for weaning [16–20]. The criteria for eligibility for the weaning trial included resolution or improvement in the disease that led to commencement of mechanical ventilation; FiO_2_ ≤ 30%; pressure support ≤ 10 cmH_2_O; PEEP ≤ 5 cmH_2_O; tidal volume ≥ 5 ml/kg; respiratory rate < 35 breaths/min; requirement of low inotropic or vasopressor support; light level of sedation (Richmond Agitation Sedation Score between − 2 and + 2) [21]; ability to cough; and absence of excessive bronchial secretions. The weaning trial comprised 60 min of pressure support of 0 or ≤ 10 cmH_2_O if required, to achieve a respiratory rate < 35 breaths/min and a tidal volume of ≥ 5 ml/kg [16]. Positive end expiratory pressure remained at 5 cmH_2_O during the trial.

The weaning trial was considered failed if the following criteria were observed during the trial: excessive diaphoresis, Richmond Agitation and Sedation Score of ≤ − 3 or ≥ + 3, evidence of increasing respiratory effort (dyspnea, increased accessory muscle activity, thoraco-abdominal paradoxical movement and facial signs of distress), arterial oxygen partial pressure ≤ 60 mmHg or oxygen saturation < 90% on fractional inspired oxygen ≥ 0.4, CO_2_ partial arterial pressure > 50 mmHg, or an increase in CO_2_ partial arterial pressure by ≥ 8 mmHg, pH < 7.32 or a decrease in pH by ≥ 0.07 pH units, RSBI > 105 breath/min/l, respiratory rate > 35 breaths/min, heart rate > 140 beats/min or increased by ≥ 20%, systolic blood pressure > 180 mmHg or increased by ≥ 20%, systolic blood pressure < 90 mmHg or new cardiac arrhythmias. After the completion of the weaning trial, the treating intensivist reviewed the participant for final assessment before extubation. The treating intensivist was blinded to the ultrasound findings. The decision whether to extubate the participant was determined by the treating intensivist irrespective of the weaning trial outcome. Failed extubation was defined as the requirement for re-intubation, non-invasive ventilatory support or death within 48 h after extubation.

### Cardiac ultrasound

**Fig. 2:**
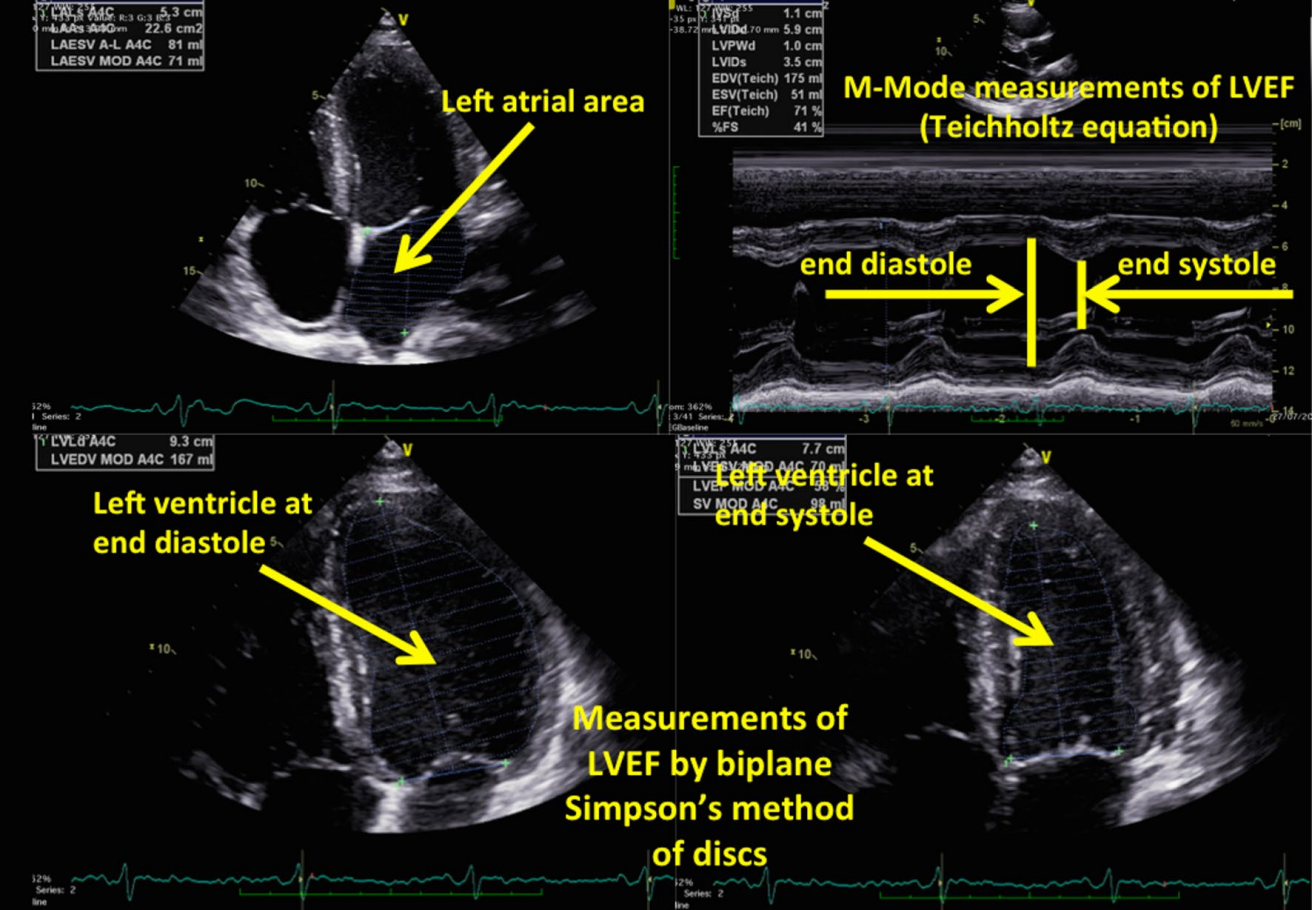
Echocardiographic assessment of left ventricular systolic function (Simpson’s method and Techoltz equation) as defined by Lang et al. [22]

**Fig. 3:**
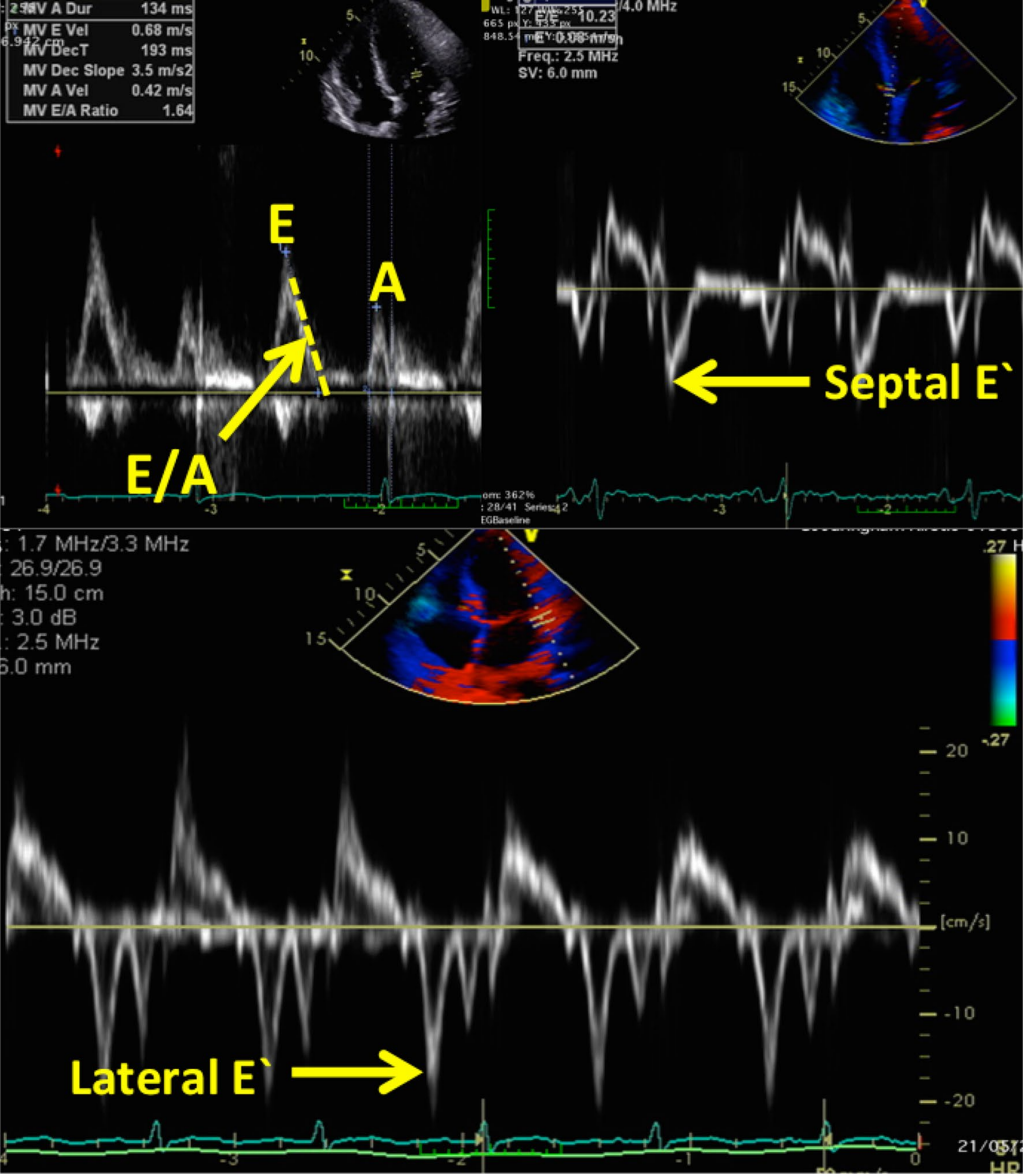
Examples of Doppler assessments used for assessment of left ventricular diastolic function (trans-mitral inflow and tissue Doppler assessment of the septal and lateral mitral valve annulus). *A*, late diastolic trans-mitral flow velocity wave, *E* early diastolic trans-mitral flow velocity wave, *E′*, early diastolic mitral annulus velocity

**Fig. 4:**
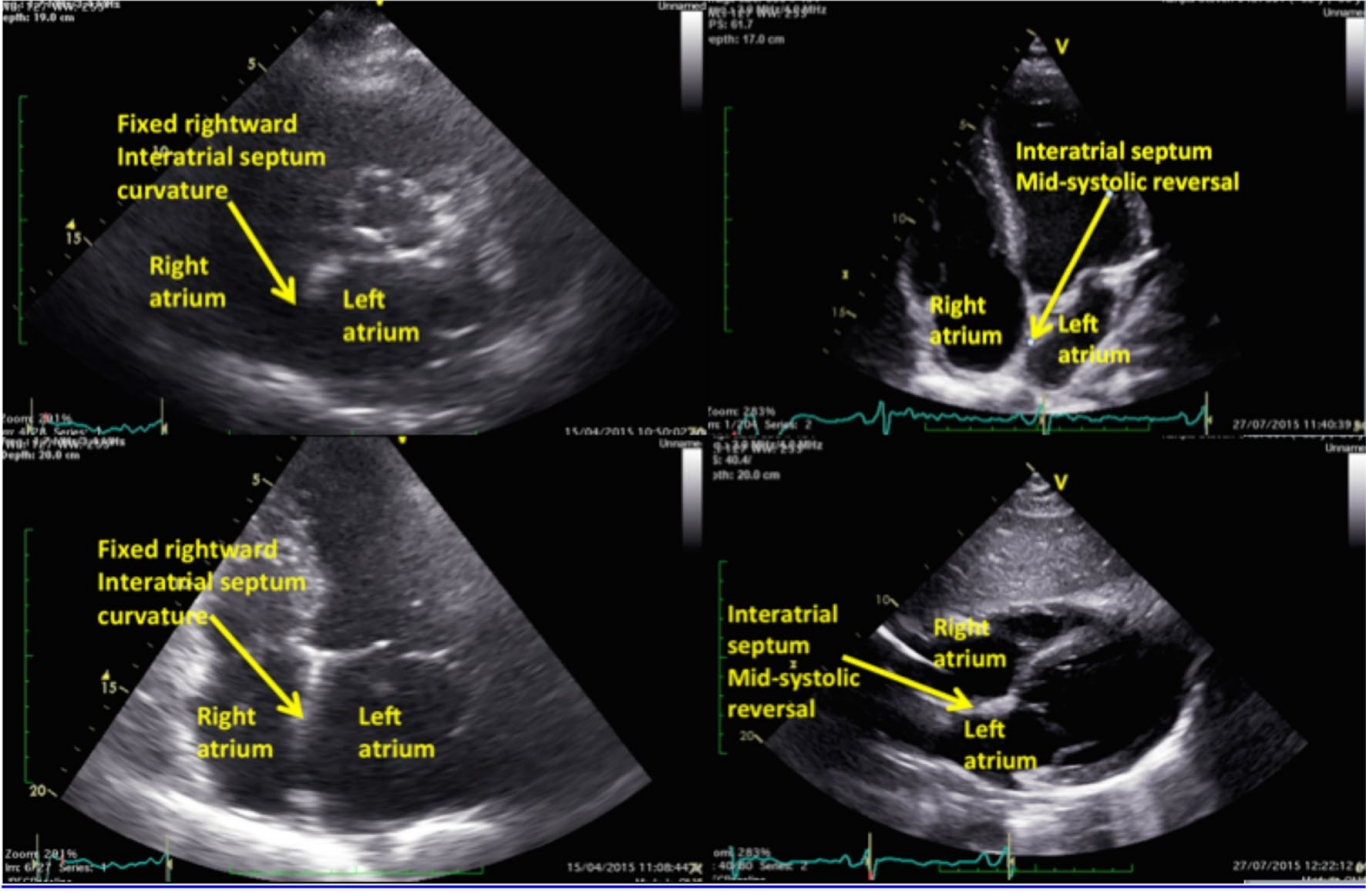
Interatrial septal movements during the cardiac cycle. Fixed rightward interatrial septum curvature is bowing of the interatrial septal to right throughout the cardiac cycle, and mid-systolic reversal is reversal of the septum from right to left during mid-systole

Ultrasound was performed by the primary researcher (KH), who held a recognized ultrasound certification and competence in TTE and an appropriate training and experience in lung and diaphragm ultrasound. The ultrasound machine used was a Vivid E9 (GE Healthcare, 9900 Innovation Drive, Wauwatosa, WI 53226, USA). Cardiac ultrasound was performed using sector phased array 3Sc-RS cardiac probe (frequency range of 1.3–4.0 MHz). The left ventricular systolic function was assessed by measuring the ejection fraction, using bi-plane Simpson’s method of discs, and/or acquisition 2-D and M-Mode measurements at the base of the left ventricle (Teichholtz equation), depending on the quality of the images, as per the recommendations from the American Society of Echocardiography (ASE) [22] (Fig. 2). Left ventricular diastolic function was assessed using left atrial area in participants who did not have haemodynamically significant mitral regurgitation or atrial fibrillation (Fig. 2), early diastolic trans-mitral flow velocity wave (*E*), late diastolic trans-mitral flow velocity wave (*A*), early diastolic mitral annulus velocity *E′* (average septal and lateral *E′*), *E*/*A*, *E* deceleration time, *E/E′* (Fig. 3). Additionally, the interatrial septal shape and movement was measured from the parasternal and subcostal short axis, and apical four chamber views, and used as a marker of left atrial filling pressure (Fig. 4) [23–25]. Fixed interatrial septal curvature to the right throughout the cardiac cycle is predictive of a pulmonary artery occlusion pressure > 15 mmHg, while reversal of the septum from right to left during mid-systole (referred to as “mid-systolic reversal”) is predictive of a pulmonary artery occlusion pressure of 10–15 mmHg. Marked septal movement and buckling during mid-systole (referred to as “mid-systolic buckling”) predicts a pulmonary artery occlusion pressure of < 5 mmHg [24].

### Lung and diaphragm ultrasound

Lung and diaphragm ultrasound was performed using the curved array 4C-RS probe (frequency range of 1.8–6.0 MHz).

#### Lung ultrasound

**Fig. 5:**
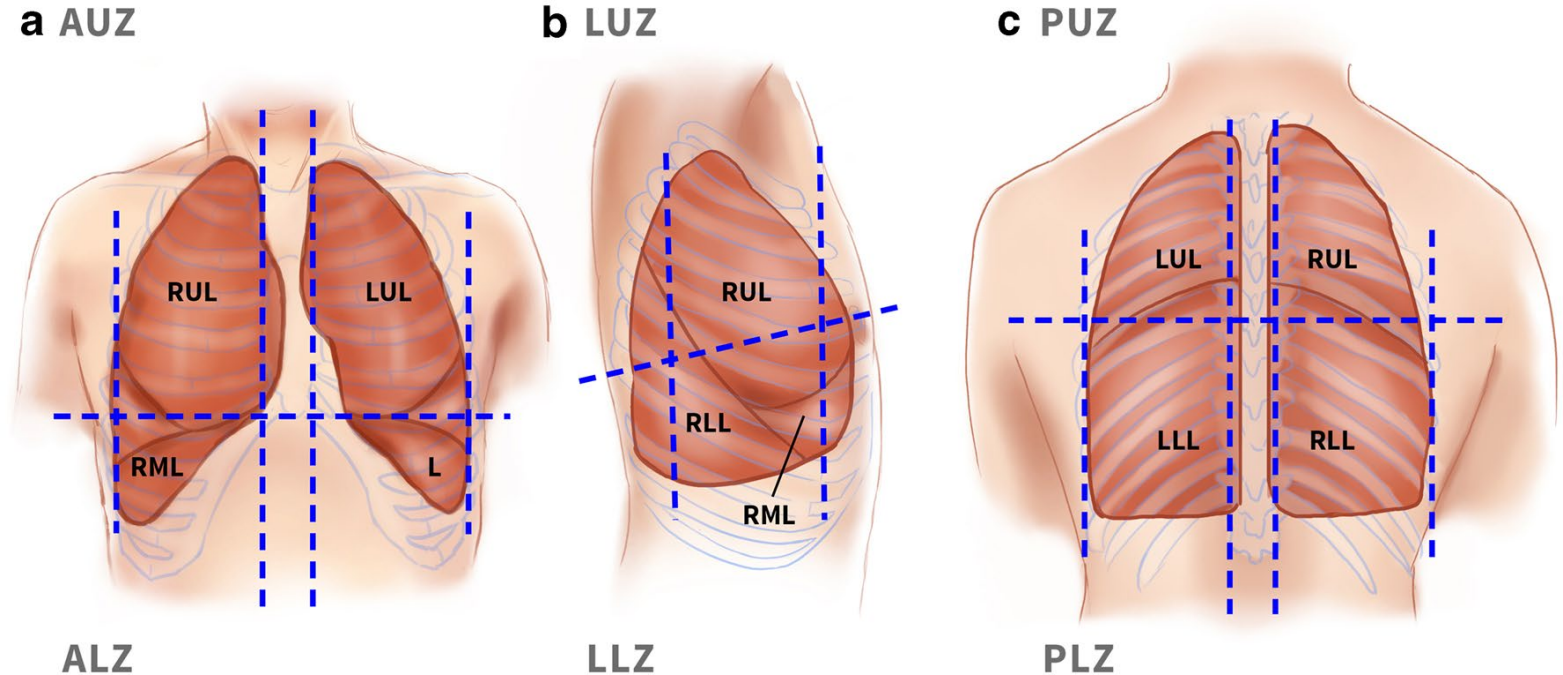
Surface anatomy of the regions of the lung. Ultrasound surface anatomy: the parasternal, anterior axillary and posterior axillary lines divide the chest wall into anterior, lateral and posterior regions. **a** The anterior region, delineated superiorly by the clavicle, inferiorly by the liver or the spleen, medially by parasternal line and laterally by the anterior axillary line. The anterior region is further divided by an arbitrary line to anterior upper zone and anterior lower zone. **b** The lateral region, delineated superiorly by the axilla, inferiorly by the liver or the spleen, anteriorly by the anterior axillary line and posteriorly by the posterior axillary line. An arbitrary line further divides the lateral region to lateral upper zone and lateral lower zone. **c** The posterior region, delineated by the posterior axillary line laterally and paravertebral line medially. A horizontal arbitrary line at the tip of the scapula further divides the posterior region to upper and lower zone. *ALZ* anterior lower zone, *AUZ* anterior upper zone, *L* lingula, *LLL* left lower lobe, *LLZ* left lower zone, *LUL* left upper lobe, *PLZ* posterior lower zone, *PUZ* posterior upper zone, *RLL* right lower lobe, *RML* right middle lobe, *RUL* right upper lobe

**Fig. 6:**
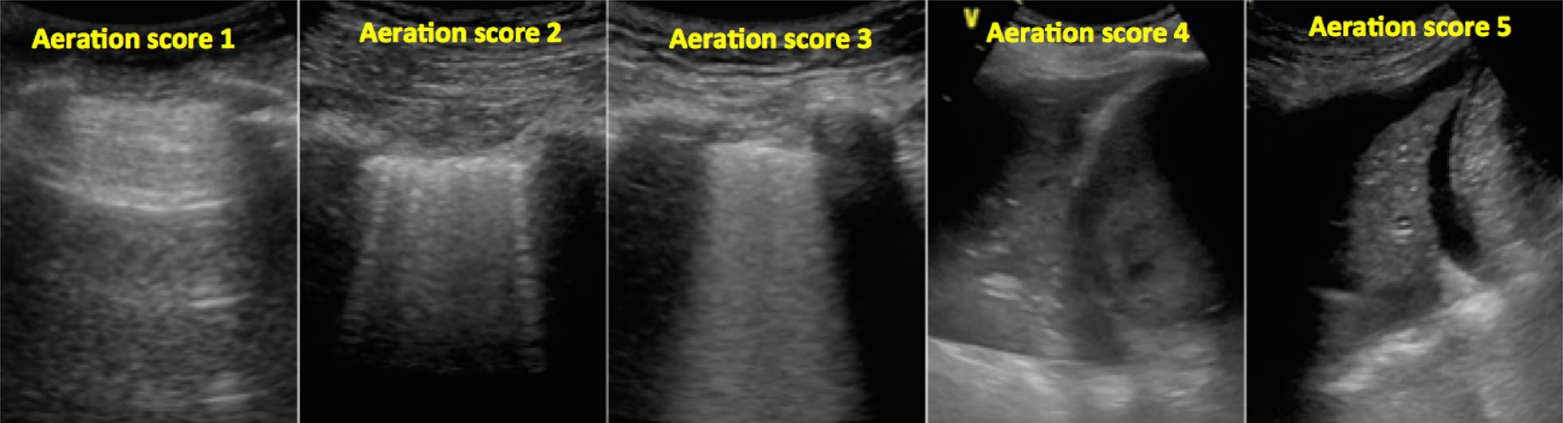
Lung ultrasound aeration scoring. The pattern and the extent of aeration defect is quantified as follows: A normally aerated or fewer than 3 B-lines scored 1; multiple discrete B-lines scored 2; multiple and fused B-lines scored 3; unaerated consolidated lung scored 4; unaerated consolidation with pleural effusion scored 5. Hence, the maximum score for each half was 5 and the maximum score for the posterior zone was 5

Lung ultrasound was performed on all participants in a semi-recumbent position at 30°–50° using the six-zone method of each lung as described by Gargani [26] and Volpicelli [27]. The surface anatomy of the lung lobes and lung ultrasound zones are shown in Fig. 5. Each intercostal space was carefully examined by sliding the probe longitudinally over the chest in each region. The exception was the posterior upper zone, where access was limited in mechanically ventilated patients. Moreover, examination of the posterior upper zone was considered to add little information, as the anterior ultrasound examination provided adequate details about the upper lobe of the lung. An aeration score was assigned to each zone based on the presence and degree of aeration loss [11], as shown in Fig. 6. Score 1 represented a normally aerated zone with the absence of/fewer than three B-lines. Score 2 represented moderately aerated zone with three or more discrete B-lines; score 3 represented severe loss of aeration with multiple and fused B-lines; score 4 represented consolidation; score 5 represented consolidation with pleural effusion. Hence, the scoring value of each zone varied from a minimum of one score to a maximum of five scores depending on the degree of the aeration loss. The sum of all zone’s score represented the global lung ultrasound score. Bilateral presence of three or more B-lines in two zones or more anteriorly and/or laterally denoted interstitial syndrome suggestive of cardiogenic pulmonary oedema [28], and the score of all zones denoted the overall aeration state.

#### Diaphragmatic ultrasound

**Fig. 7:**
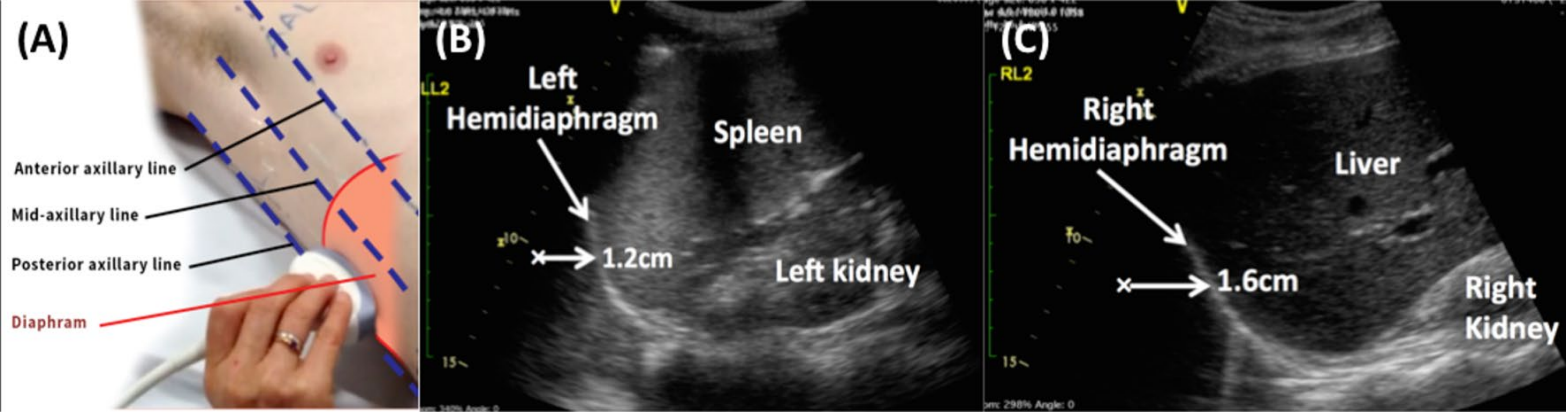
Ultrasound technique and images of the diaphragm. **A** The location of probe placement on the chest wall for assessment of diaphragm movement. The probe is placed laterally and perpendicularly on the lateral chest wall on the lower intercostal spaces between mid- and posterior axillary line. **B**, **C** The ultrasound images of the left and the right hemidiaphragm and the measurement of the diaphragmatic excursion from end of end inspiration to end expiration

The lateral approach was used to image the diaphragm, with the participants in a supine and head-up position at 30°–50° (Fig. 7). The same landmarks and techniques were applied on the right and left hemidiaphragm. The probe was placed laterally and perpendicularly on the lower intercostal spaces of the lateral chest wall between mid- and posterior axillary line [29, 30]. The direction of the ultrasound beam was perpendicular or near perpendicular to the craniocaudal axis during breathing. This provided adequate visualization of the maximum displacement of the diaphragm during breathing. The images of the hemidiaphragm movement were recorded for at least three consecutive respiratory cycles. The range of movement between 2 points (end of inspiration and end of expiration) for all recorded cycles was measured and averaged. The images during forced breaths, sighs and during suctioning were excluded. Diaphragmatic movement of greater than 1 cm represented normal function [31]. Unilateral dysfunction was defined when a unilateral hemidiaphragm movement was less than 1.0 cm; bilateral dysfunction was defined as movement of less than 1.0 cm in both hemidiaphragms.

### Reliability of imaging

Reliability of ultrasound measurements of the heart, lungs and diaphragm was assessed by measuring the interobserver variability between two observers with appropriate training and experience in these measurements. Digital images of at least three cardiac cycles and three respiratory cycles for each standard view were assessed off-line using Synapse Cardiovascular software (Fujifilm, Akasaka, Minato, Tokyo, Japan). The mean difference and limits of agreement (± 2 SDs of the difference) between the two observers was recorded. We considered the agreement between observers to be acceptable if the limits of agreement were less than 20% of the mean value of the variable being measured for continuous data.

### Statistical analysis

Patient characteristics were summarized as *n* and % for categorical variables and as median and interquartile range for continuous variables. Comparison of quantitative variables was performed using independent samples *t* test or Mann–Whitney test. Comparison of categorical data was performed using Fisher’s exact test. In order to determine the key predictors of successful extubation, odds ratio (OR) was obtained for each variable using univariate binary logistic regression. Due to the small sample size and therefore increased risk of Type II error, and also to reduce risk of Type I error due to multiple pairwise comparisons, the *p* value for significance is defined as *p* < 0.01. As the effect size for the ultrasound endpoints was not known, recruitment was based on a time period not exceeding 2 years, and with a maximum of 80 participants for the pilot study. All analyses were performed in SPSS version 23 (IBM Corp.).

## Results

Of the 58 participants screened during the study period, five did not meet inclusion criteria: two participants had a tracheostomy, one participant died from septic shock and two participants were extubated without informing the investigators, and were not recruited. This left 53 participants for data analysis, two-thirds of whom were male. Sepsis (38%) was the most common admission diagnosis. Other diagnoses included post-surgical (gastrointestinal, vascular, musculoskeletal and burns) (15%), drug overdose (13%), gastrointestinal disease (decompensated chronic liver disease, acute pancreatitis, upper gastrointestinal bleeding) (12%), cardiac arrest (8%), neurological disease (status epilepticus, motor neuron disease, encephalitis) (6%) acute cardiogenic pulmonary oedema (4%) and metabolic disorder (hyponatremia and diabetic ketoacidosis) (4%).

All 53 recruited patients followed the weaning trial protocol. All 53 patients received PEEP of 5 cmH_2_O. Pressure support received during weaning trial was zero (60%), 5–7 cmH_2_O (15%) and 8–10 cmH_2_O (25%). Fifty patients passed the weaning trial (94.4%) and three participants failed the weaning trial (5.6%). The treating intensivists, however, decided to extubate all 53 patients. Of the three participants who were extubated despite failing the weaning trial, two participants (systolic blood pressure of 200 mmHg and agitation score of + 3) were successfully extubated and one participant (tidal volume 4 ml/kg) failed extubation. Of the 50 participants who passed the weaning trial and were extubated, 40 (80%) participants were successfully extubated. Of the total 53 participants, 11 (20.7%) participants failed extubation over the subsequent 48 h. Two of the 11 (18%) participants that failed extubation were re-intubated and the remaining nine (82%) received non-invasive ventilation. The reasons for failure of extubation as reported by the treating team were pulmonary oedema [7], hospital acquired pneumonia [1], post-operative pain and agitation [1], and deconditioning with insufficient respiratory effort [2].

The participant demographic data are shown in Table 1. Variables that were associated with failed extubation included known pre-existing ischemic heart disease (OR 6.41, 95% CI 1.53–26.88, *p* = 0.01), pre-existing diabetes mellitus (OR 4.40, 95% CI 1.08–17.79, *p* = 0.03), higher respiratory rate (OR 1.14 95% CI 1.01–1.27, *p* = 0.02) and RSBI (OR 1.03, 95% CI 1.00–1.06, *p* = 0.03). BNP was not associated with success or failure of extubation. There was no group separation for ICU and hospital length of stay. Two participants who failed extubation died and three participants who were successfully extubated died during their hospitalization.

**Table 1:** Clinical and biochemical variables of failed and successful extubation groups

	Failed extubation (*n* = 11)	Successful extubation (*n* = 42)	Unadjusted OR (95% CI)	*p* value
Characteristics				
Male *n* (%)	10 (90.9)	24 (57.1)	0.13 (0.01–1.14)	0.06
Age, years median (IQR)	77.0 (65.0–81.0)	63.5 (45.7–73.3.0)	1.05 (0.99–1.11)	0.06
Ideal body weight (kg) median (IQR)	61.0 (54.7–63.9)	62.3 (54.6–66.2)	0.96 (0.87–1.06)	0.44
BMI kg/m^2^ median (IQR)	27.0 (23.3–35.1)	27.0 (24.1–31.0)	1.0 (0.9–1.11)	0.95
Ischaemic heart disease *n* (%)	7 (63.6)	9 (21.4)	6.41 (1.53–26.88)	0.01
Heart failure *n* (%)	3 (27.3)	5 (11.9)	2.77 (0.54–14.05)	0.21
Diabetes mellitus *n* (%)	6 (54.5)	9 (21.4)	4.40 (1.08–17.79)	0.03
COPD *n* (%)	3 (27.3)	6 (14.3)	2.25 (0.46–10.96)	0.31
ICU severity score				
APACHE II median (IQR)	20.0 (17–23)	20.0 (15.0–23.3)	1.06 (0.94–1.20	0.31
APACHE III median (IQR)	82.0 (61.0–97.0)	74.5 (60.5–89.5)	1.02 (0.99–1.04)	0.21
SAPS II median (IQR)	42.0 (33.0–46.0)	46.0 (36.0–57.3)	0.99 (0.94–1.04)	0.72
Weaning trial				
Respiratory rate (breath/min) median (IQR)	24 (18–30)	18 (15–23)	1.14 (1.01–1.27)	0.02
Tidal volume (l) median (IQR)	0.48 (0.39–0.58)	0.51 (0.41–0.75)	0.11 (0.00–5.76)	0.27
Pressure support (cmH_2_O) median (IQR)	0.0 (0.0–10.0)	0.0 (0.0–7.2)	1.04 (0.89–1.22)	0.58
ICU outcomes				
Extubation failure *n* (%)				
ICU LOS (day) median (IQR)	10.5 (9.7–17.5)	7.9 (5.5–13.0)	1.14 (0.99–1.25)	0.06
Time from extubation to ICU discharge (days) median (IQR)	4.1 (3.1–7.1)	1.9 (1.0–5.1)	1.13 (0.95–1.34)	0.14
Hospital LOS (day) median (IQR)	19.2 (16.1–33.1)	16.4 (11.2–24.1)	1.00 (0.96–1.04)	0.71
In-hospital mortality *n* (%)	2 (18.2)	3 (7.1)	0.34 (0.05–2.38)	0.28
Duration of MV (h) median (IQR)	175.0 (99.1–238.5)	130.6 (93.7–180.5)	1.00 (0.99–1.01)	0.11

*APACHE* acute physiology age and chronic health evaluation, *BMI* body mass index, *BNP* B-type natriuretic peptide, *COAD* chronic obstructive airway disease, *H LOS* hospital length of stay, *ICU LOS* intensive care unit length of stay, *SAPS* simplified acute physiology score, *IQR* interquartile range

The cardiac, lung and diaphragm ultrasound findings, RSBI and BNP are shown in Table 2. Cardiac ultrasound was un-interpretable in one participant (who was successfully extubated). Failed extubation was associated with echocardiographic markers of left ventricular diastolic dysfunction, including raised left atrial pressure indicated by interatrial septal fixed rightward curvature (OR 12.95, 95% CI 2.73–61.41, *p* = 0.001), higher *E/E′* (OR 1.27, 95% CI 1.05–1.54, *p* = 0.01) and larger left atrial area (OR 1.14, 95% CI 1.02–1.28, *p* = 0.02).

**Table 2:** Clinical, echocardiographic and respiratory ultrasound variables

Variable	Failed extubation (*n* = 11)	Successful extubation (*n* = 42)	Unadjusted OR (95% CI)	*p* value
RSBI (breath/min/l) median (IQR)	48.0 (35.1–72.0)	33.5 (25.6–55.4)	1.03 (1.00–1.06)	0.03
BNP (pmole/l) median (IQR)	372 (152–1620)	233 (95–475)	1.0 (0.99–1.00)	0.72
Echocardiographic variables				
LVEF (%) median (IQR)	50 (45–55)	65 (53–65)	0.96 (0.91–1.0)	0.1
*E* median (IQR)	0.87 (0.68–1.04)	0.83 (0.68–0.99)	5.43 (0.35–85.2)	0.22
*E*/*A* median (IQR)	1.20 (0.60–1.64)	1.13 (0.85–1.36)	0.99 (0.38–2.59)	0.99
EDT median (IQR)	175 (125–259)	180 (155–217)	0.99 (0.98–1.01)	0.87
*E′* median (IQR)	0.07 (0.05–0.11)	0.10 (0.08–0.12)	0.00 (0.00–1.36)	0.052
*E/E′* median (IQR)	10.9 (8.7–18.0)	7.7 (6.4–10.6)	1.27 (1.05–1.54)	0.01
LAA median (IQR)	25.0 (19.3–32.0)	20.0 (18.0–23.7)	1.14 (1.02–1.28)	0.02
IAS fixed curvature *n* (%)	7 (72.7)	8 (17.1)	12.95 (2.73–61.41)	0.001
Lung ultrasound				
Aeration score of left and right anterior and lateral region (upper and lower zones) median (IQR)	17 (8–20)	11 (8–13)	1.41 (1.01–1.82)	0.007
Aeration score of left and right lungs total median (IQR)	22 (13–28)	18 (13–21)	1.12 (0.99–1.27)	0.06
Diaphragmatic dysfunction *n* (%)				
No diaphragmatic dysfunction	5 (45.5)	24 (57.1)		0.53
Hemidiaphragm dysfunction	4 (36.4)	15 (35.7)	1.28 (0.29–5.53)	0.26
Diaphragm dysfunction	2 (18.2)	3 (7.1)	3.2 (0.41–24.41)	0.49

Normal values of the parameters listed in the table are as follows: RSBI: < 105, BNP < 29 pmol/l, LVEF < 50%, *E* 73 ± 19 cm/s, *A* 69 ± 17 cm/s, *E*/*A* 1.20 ± 0.20, DT 192 ± 40 ms, *E′* 12 ± 4 cm/s, *E*/*E*′ 10 ± 2, hemidiaphragm excursion ≥ 1.0 cm

IAS: interatrial septum; IQR: interquartile range; LAA: left atrial area; LVE/*E*: left ventricular early diastolic trans-mitral flow velocity; *E* wave: early diastolic mitral annulus velocity *E′* ratio; LVEF: left ventricular ejection fraction; OR: odds ratio, RSBI: rapid shallow breathing index

Higher loss of aeration score of the anterior and the lateral lung regions was associated with failed extubation (OR 1.41, 95% CI 1.01–1.82, *p* = 0.007). The magnitude of diaphragmatic movement was not associated with success or failure of extubation.

The interobserver variation showed acceptable agreement. The limits of agreement, using a Bland–Altman approach, were less than 20% of the mean value for all comparisons. There were no critical findings that required immediate disclosure to the treating team for the safety of the participants.

## Discussion

Of the clinical and ultrasound indicators in this pilot study, we determined that the most likely parameters which could be useful to help predict failed extubation are those which are related to diastolic dysfunction (left atrial area, *E/E′*, interatrial septal rightward fixed curvature), loss of aeration score of the left and right anterior and lateral regions, and increased RSBI. Interatrial septal fixed curvature and lung ultrasound findings appear promising and may aid in decision-making for extubation, as they are easily and quickly assessed, even in operators with limited echocardiographic experience. However, these results need to be verified in a larger study. The value of combined heart and lung ultrasound is that the physician extends their clinical evaluation to both the cardiac and respiratory systems to identify pathology. In some participants, pathology was confined to either the heart or lungs, and in others, pathology existed in both systems. Further, adding lung findings to heart ultrasound can act as a measure of severity. Systolic or diastolic failure patterns on heart ultrasound, for example, will have a greater severity if there is evidence of interstitial oedema on lung ultrasound, and more so is present in both upper and lower lobes.

Interestingly, participants who were older with diabetes and ischemic heart disease had greater incidence of extubation failure when compared to those who were successful. Such association between weaning failure and diabetes was also reported in a similar study [10]. Although unclear, the association is possibly attributed to left ventricular diastolic failure. This perhaps suggests that older patients, patients with diabetes and or ischaemic heart disease may most benefit from bedside cardiac ultrasound as an adjunct to prediction of their candidacy for ventilatory weaning and endotracheal extubation.

Our study particularly focused on imaging the heart, lung and the diaphragm although only during the weaning trial just before extubation. We believe that ultrasound may provide an opportunity for the intensivist to optimize the patient’s cardiac and respiratory function before extubation, and perhaps modify the weaning mechanism, such as transitioning those at risk to non-invasive ventilation before discontinuing the ventilatory support [32, 33].

Our data do not allow us to determine why diastolic dysfunction is associated with weaning failure. However, it is reasonable to assume that patients with raised left atrial pressure may be more likely to develop pulmonary venous congestion as the hemodynamic stress associated with weaning increases. A study by Caille et al. suggested that patients are more likely to fail weaning when their left ventricular ejection fraction is lower than 35% and *E/E′* is greater than 7.8 [8]. Conversely, Moschietto et al. [10] showed that unlike *E/E′*, ejection fraction had no impact on weaning outcome. Recent published data have demonstrated a positive association between the echocardiographic measurements of diastolic failure, in particular *E/E′*, and weaning failure [8–10, 34]. *E/E′* is a marker of left atrial pressure, and that of left ventricular diastolic pressure. *E/E′* correlated well with pulmonary artery occlusion pressure [35, 36]. However, this correlation in the operating room setting was debatable [25]. *E/E′* range of cut-off values of 7–14 has been suggested for determining patients at risk of failing weaning from mechanical ventilation [8, 9, 34]. In another study, a higher cut-off value of 14.5 during a spontaneous breathing trial had a sensitivity of 75% and specificity of 95.8% for predicting weaning failure [10].

Fixed rightward curvature of interatrial septum was a prominent finding in the failed extubation group. Assessment of interatrial septal movement is an easy technique to perform to obtain a visual estimation of the left atrial pressure state. Royse et al. [24] found that pulmonary artery occlusion pressure greater than 15 mmHg corresponded to fixed rightward curvature of the interatrial septal movement throughout the cardiac cycle. The findings were supported by an earlier study, where the shape and movement of the interatrial septum were dependent on the gradient between the atria [23]. Interatrial septal movement is easily obtained from a 2-dimensional view of any standard parasternal short axis, apical 4-chamber or subcostal 4-chamber or subcostal short axis.

Left atrial enlargement is an indicator of severity and chronicity of diastolic dysfunction and is associated with increased left atrial pressure [22]. However, left atrial enlargement is non-specific and it is influenced by other factors such as hemodynamically significant mitral valve pathology and atrial fibrillation [37]. There were two participants from the successful group who had atrial flutter during imaging but all participants in the failed group were in sinus rhythm.

Lung ultrasound is a non-invasive and easily performed bedside test. It has clinical and practical advantages over conventional chest radiography [27, 38]. Besides its diagnostic capability, lung ultrasound is useful for monitoring lung aeration during prone positioning [39]. The pattern, location and the extent of loss of aeration on ultrasound identify the pathological process and hence the diagnosis of the respiratory disorder. The loss of aeration scoring method used in this study provides a quantification of the degree of aeration defect, which may be useful in assessing the type, distribution and degree of lung injury. A similar scoring system was used by Soummer et al. [11] for predicting post-extubation distress during a spontaneous breathing trial. Pleural effusion was included in our scoring system, which added to the severity of aeration loss. In this study, an association with worse aeration was found in participants who failed extubation. Moreover, the association was stronger with significantly higher loss of aeration score, when the antero-lateral regions of both lungs were included. This finding may translate to a higher incidence of pulmonary oedema in the failed extubation group, consistent with cardiac failure [26, 28].

Mechanical ventilation may induce diaphragmatic dysfunction from muscle disuse, particularly in prolonged mechanical ventilation [40]. Diaphragmatic disuse leads to protein oxidation via activation of autophagy, lysosome pathway and skeletal muscle proteolysis [41], which subsequently alters the diaphragm contractile property. In a study by Kim et al. [13], the prevalence of diaphragmatic dysfunction was 29% in critically ill patients with no history of neuromuscular or diaphragmatic diseases. In this study, the cut-off diaphragm excursion value for extubation failure was 1.4 cm for the right hemidiaphragm and 1.2 cm for the left hemidiaphragm [13]. Further, patients with diaphragmatic dysfunction had a longer ventilation time, 401 h vs. 90 h in patients with normal diaphragm. In another study, liver and spleen displacement as a surrogate for respiratory sufficiency, in particular diaphragmatic movement, has been shown as predictors for successful extubation when compared to RSBI, spontaneous tidal volume or maximum inspiratory pressure [42]. In contrast to the published evidence about the diaphragmatic role in respiratory weaning outcome, we did not find an association between diaphragm dysfunction and extubation failure. However, due to the small sample size in our study, the role of the diaphragm in weaning needs further exploration.

On current practice, the prediction of successful weaning and readiness for extubation is assessed using clinical and biochemical parameters including the degree of respiratory support, respiratory rate, tidal volume, RSBI and arterial blood gas analysis. RSBI is a surrogate marker of quality of breathing during weaning from mechanical ventilation. It is defined as the ratio between respiratory rate and tidal volume where a value greater than 105 breaths/min/l predicts patients at high risk of failing weaning [14, 43]. However, weaning failure has been associated with RSBI of lower than recommended values [11]. In our study, higher RSBI was weakly associated with failed extubation. BNP is a biochemical marker of cardiac dysfunction. It is a 32-amino acid peptide released into the plasma because of cardiomyocytes over-stretch. The role of BNP in weaning from mechanical ventilation is controversial [7, 11, 44]. In our study, there was a weak association with elevated BNP and extubation failure.

This is a pilot study, and therefore it can only be used to identify potentially useful parameters to aid clinical decision-making. The study was based on convenience sampling, which was subject to the availability of the research sonographer. In contrast to some previously published studies, our study protocol did not interfere with the clinician’s extubation decision, and all participants proceeded to extubation regardless of weaning trial outcome. In the lung ultrasound, we did not image the postero-superior areas due to difficult windows in supine ventilated patients, and the upper lobe can be visualized from the anterior approach. It is possible, however, that some upper lobe pathology may have been missed. The results of this study can be further tested by a larger prospective study in a high-risk population that perhaps are older with history of diabetes and/or ischemic heart disease and with a clinical diagnosis of cardiac.

## Conclusions

Failed extubation in mechanically ventilated patients is more prevalent if markers of left ventricular diastolic dysfunction and loss of lung aeration are present.

## Abbreviations

A: late diastolic trans-mitral flow velocity wave
BNP: B-type natriuretic peptide
*E*: early diastolic trans-mitral flow velocity wave
*E/E′*: early diastolic trans-mitral flow velocity wave/early diastolic mitral annulus velocity
ICU: intensive care unit
*E*/*A*: early diastolic trans-mitral flow velocity wave/late diastolic trans-mitral flow velocity wave
PEEP: positive end expiratory pressure
RSBI: rapid shallow breathing index
TTE: transthoracic echocardiogram
